# Reply: matters arising to impact of a POCUS-first versus CT-first approach on emergency department length of stay and time to surgical consultation in patients with acute cholecystitis: a retrospective study

**DOI:** 10.1186/s13049-025-01375-6

**Published:** 2025-04-15

**Authors:** Chien-Tai Huang, Wan-Ching Lien

**Affiliations:** 1 Department of Emergency Medicine, National Taiwan University Hospital Hsin-Chu Branch, Hsinchu City, Taiwan; 2 Department of Emergency Medicine, College of Medicine, National Taiwan University, Taipei, Taiwan; 3 Department of Emergency Medicine, National Taiwan University Hospital, No. 7, Chung-Shan South Road, Taipei, 100 Taiwan

We appreciate the opportunity to receive thoughtful feedback from our readers [1]. We acknowledge the importance of assessing the effectiveness of point-of-care ultrasound (PoCUS) in patients with Grade II-III acute cholecystitis (AC), as these cases exhibit more pronounced inflammation and a higher risk of complications. However, only 85 patients (4%) in our study population were classified as Grade II-III, which was insufficient to draw a definitive conclusion on the effectiveness of PoCUS. Additionally, computed tomography (CT) has a higher diagnostic yield than abdominal ultrasound for detecting emphysematous and gangrenous cholecystitis, both of which are classified as Grade II according to the TG13 severity grading system [2, 3].

The readers questioned why CT was used as a mandatory diagnostic tool in our study. A recent meta-analysis reported that ultrasound has a pooled sensitivity of 71%, specificity of 85%, and accuracy of 83% for diagnosing AC [4]. To enable a more reliable comparison between PoCUS and CT and to minimize false-positive cases, we included patients with CT-confirmed AC in our study. Furthermore, at our institution, surgeons rely on CT findings for clinical decision-making, and as a result, CT is performed in all patients with AC.

The readers also questioned why, despite PoCUS being performed within 60 min, the median time to surgical consultation and ED length of stay (ED-LOS) remained prolonged, suggesting that external factors significantly influenced patient flow management. This can be attributed to our hospital being an academic medical center, where ED admissions account for only 18% of total hospital admissions. A shortage of hospital beds has a major impact on patient flow, and bottlenecks in patient output contribute to delays from diagnosis to ED discharge. The effectiveness of PoCUS would be more pronounced in improving patient admission flow.

Notably, conducting PoCUS within 60 min resulted in a time savings of 22.4 h of ED-LOS compared to cases where PoCUS was performed after 60 min. In contrast, performing CT within 120 min was associated with a reduced ED-LOS, decreasing the ED-LOS by 12 h. These findings suggest that timely imaging studies reflect the proactivity of attending physicians, ultimately improving ED patient flow.

Moreover, the readers inquired about how the timing of PoCUS and CT was determined. As shown in Table 2 [5] Impact of a POCUS-first versus CT-first approach on emergency department length of stay and time to surgical consultation in patients with acute cholecystitis: a retrospectivestudy., we selected the optimal timing based on the coefficients in the linear regression model, aiming to balance both the reduction in ED-LOS and the time to surgical consultation.

We appreciate the opportunity to address these important points and further clarify our findings. Further studies will be needed to validate the effectiveness of PoCUS and CT in diagnosing of Grade II-III AC.

## Data Availability

No datasets were generated or analysed during the current study.
